# Age‐Dependent Plasticity in Hydraulics and Allocation of K, Si, and Starch in Moso Bamboo (*Phyllostachys pubescens*)

**DOI:** 10.1111/ppl.70745

**Published:** 2026-01-19

**Authors:** Yan Xiang, Yasuhiro Utsumi, Shinya Koga, Tomonori Kume, Satoshi Nagai, Maojiao Yang

**Affiliations:** ^1^ Faculty of Agriculture University Forest, School of Agriculture, Kyushu University Fukuoka Japan; ^2^ Hyogo Prefectural Technology Center for Agriculture Forestry and Fisheries Shiso Japan; ^3^ Graduate School of Bioresource and Bioenvironmental Sciences Kyushu University Fukuoka Japan

**Keywords:** cryo‐scanning electron microscopy, dye tracer, Moso bamboo, relative loss of potential conductivity, sap flux density

## Abstract

Moso bamboo ( *Phyllostachys pubescens* ), a fast‐growing and potentially invasive species, exhibits culm‐age heterogeneity in structure and physiology; however, its water‐use strategies in relation to aging remain unclear. Thus, we aimed to examine age‐related variations in hydraulic performance, vessel integrity, and nutrient allocation in bamboo culms aged 1–5 years. Sap flux density peaked in 2‐year‐old culms, possibly reflecting the maturation of conductive tissues. However, daily sap flow rates showed no significant age‐dependent differences. Dye tracing and cryo‐scanning electron microscopy revealed consistent axial and radial vessel continuity and low embolism frequency across all age groups, with a relative loss of potential conductivity of approximately 10%. Elemental analysis showed reduced K concentration and delayed Si accumulation in the vessel sap with age, suggesting a physiological shift from osmotic regulation to structural reinforcement. Starch began accumulating in the third year and peaked at age four, indicating a physiological transition from resource consumption to energy storage. These coordinated transitions support sustained water transport across ages and may enhance resilience under drought and interspecific competition. Our findings revealed functional plasticity in water use and resource allocation during culm development, highlighting the physiological mechanisms that may underlie the ecological success and invasive potential of Moso bamboo.

## 1 Introduction

Moso bamboo ( *Phyllostachys pubescens* ) is widely distributed throughout Asia, growing in habitats ranging from lowland plains to mountainous regions (Li et al. 2016; Laplace et al. 2017; Chen et al. 2022). Owing to its rapid growth and favorable material properties (Yu et al. 2008), Moso bamboo is extensively used in construction, furniture manufacturing, handicrafts, and other bamboo‐related products (Dixon and Gibson 2014; van Dam et al. 2018). Its high economic value and broad range of applications have made Moso bamboo an important plant resource in many Asian countries (Bystriakova et al. 2003; Fei 2021).

In recent years, numerous Asian countries and regions, such as China, Japan, and parts of Southeast Asia, have reported the rapid invasion of Moso bamboo into adjacent plantations (e.g., cedar and cypress stands) and natural forests. This phenomenon has raised concerns regarding potential biodiversity threats and forest structural stability (Komatsu et al. 2012; Chang and Chiu 2015; Laplace et al. 2017). Moso bamboo exhibits higher transpiration rates than other tree species, enabling it to absorb large quantities of soil water and lower soil moisture levels (Kume et al. 2010; Ichihashi et al. 2015). This property can lead to water shortages or water stress in neighboring trees, raising further concerns about the ecological impacts of bamboo expansion (Wu et al. 2019). Therefore, a thorough understanding of the water physiology in Moso bamboo at different growth stages is essential for biodiversity conservation, forest management, and regional water resource regulation.

Transpiration rate is a key indicator for assessing water use by Moso bamboo. Several studies have suggested that bamboo culms of different ages significantly vary in transpiration rates, potentially because of age‐related changes in xylem anatomy (Liese and Weiner 1996). Differences in sap flux density have also been observed among culms of varying ages (Dierick et al. 2010). However, the current literature offers inconsistent conclusions. Some researchers have observed that older culms have lower transpiration rates (Zhao et al. 2017a, 2017b; Gu et al. 2019), whereas others have reported no significant differences among culms of different ages (Tsuruta et al. 2016). Additionally, certain studies have shown that 2‐ to 3‐year‐old culms transpire at significantly higher rates than 1‐ and 4‐year‐old culms (Wu et al. 2019). These discrepancies indicate the absence of a consensus on how aging influences transpiration in Moso bamboo, and the underlying causes of these differences remain unclear.

In addition, the physiological mechanisms responsible for the variation in transpiration rates among Moso bamboo of different ages are not fully understood. Some studies have proposed that increases in xylem embolism reduce transpiration (Liese and Weiner 1996; Zhao et al. 2017a, 2017b; Tsuruta et al. 2016; Liese and Kohl 2015; Mei et al. 2020), but direct measurements of embolism and its relationship with bamboo culm age are still lacking.

Although nutrients and chemical elements play crucial roles in promoting bamboo growth (Umemura and Takenaka 2014), the mechanisms underlying their transport, storage, and functional integration during culm development remain poorly understood. Age‐related variations in key biochemical constituents may contribute to differences in water regulation strategies. For instance, K^+^ plays an essential role in maintaining osmotic balance and driving water uptake in actively transpiring tissues (Talbott and Zeiger 1996; Roelfsema and Hedrich 2005; Johnson et al. 2022). By contrast, Si is often associated with structural reinforcement, which potentially enhances xylem stability and resistance to collapse or embolism, particularly in older tissues (Wang et al. 2021; Collin et al. 2012). Starch accumulation is a metabolic hallmark of the shift from active growth to energy storage and long‐term maintenance (Liese and Weiner 1996; Uchida et al. 2022). However, how these biochemical traits change with culm age and coordinate hydraulic functions remains largely unexplored.

The present study aimed to clarify three points regarding age‐dependent functional plasticity in Moso bamboo culms: (1) whether sap flux density and daily water use vary with culm age and which physiological or anatomical factors underlie these differences; (2) whether xylem network integrity and the occurrence of xylem embolism change with age, potentially reducing water‐transport efficiency; and (3) whether key biochemical traits—specifically K and Si concentrations in xylem sap and starch distribution in parenchyma—show coordinated age‐related changes that reflect shifts in water‐use strategy and metabolic function during culm development.

## 2 Materials and Methods

### 2.1 Study Site and Sample Selection

In mid‐July 2021, an experimental area measuring 21 × 27 m was established within a Moso bamboo stand in the Fukuoka Research Forest of Kyushu University (Kasuya, Fukuoka, Japan; 33°37′ N, 130°31′ E; elevation 80 m).

**FIGURE 1:**
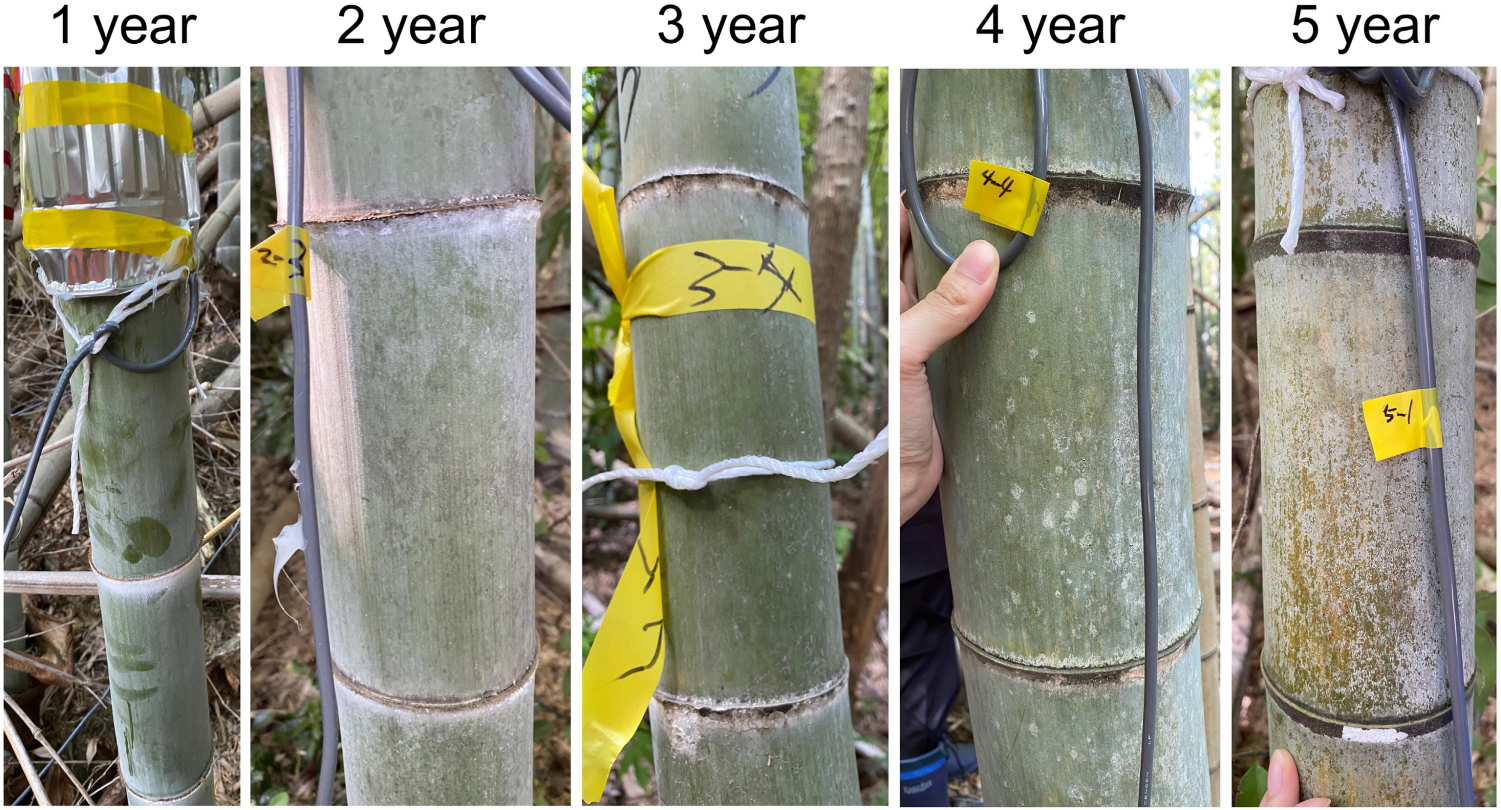
External morphological characteristics of Moso bamboo culms at different ages. The 1‐year‐old culm has a vivid green surface with thick wax deposits on the nodes, which are easily removed by touch. The 2‐year‐old culm is green with slight black speckles; node wax is still present but not easily removed. The 3‐year‐old culm is mostly green with a slight yellow tint; wax remnants are minimal, and the nodes appear darkened. The 4‐year‐old culm is green with increased yellowing and more frequent black speckles; the wax layer has disappeared, and surface erosion at the nodes is pronounced. The 5‐year‐old culm is predominantly yellow with some green areas; the epidermis is exfoliated, and the nodes have turned completely black.

Moso bamboo culms within this area were assigned an age according to the color and surface characteristics of the culm, following the methods documented by Shinohara et al. (2013) and Tsuruta et al. (2016). The assignments were validated following the criteria described by Mei et al. (2020) and Zhao et al. (2017a). Based on these criteria, five age groups (from 1 to 5) were designated (Figure 1); bamboo older than 5 years but still in healthy condition was also classified into the “5‐year‐old” group. Four bamboo culms were selected from each age group for a total of 20 culms in the experiment.

During sample collection, the diameter at breast height (DBH, cm) of each culm was measured at approximately 1.3 m above ground using a measuring tape, and the culm wall thickness (CWt, cm) was determined using Vernier calipers. The culm cross‐sectional area (1, Table 1) was calculated as follows:

(1) $$S_{A}=\left(\mathrm{DBH}\times \mathrm{CWt}-\mathrm{CWt}\times \mathrm{CWt}\right)\pi$$

**TABLE 1:** Tree height (m), diameter at breast height (DBH, cm), culm wall thickness (cm), and xylem cross‐sectional area (cm^2^) for each sample across different age groups.

Age group	Sample no.	Height (m)	DBH (cm)	Culm wall thickness (cm)	Cross‐sectional area (cm^2^)
1 year	1–1	13.5	9.13	0.82	21.35
	1–2	16.6	9.65	0.92	25.26
	1–3	14.2	9.34	0.96	25.26
	1–4	12.8	7.41	0.78	16.27
	Mean ± S.D.	14.28 ± 1.65 (ab)	8.88 ± 1.01 (a)	0.87 ± 0.08 (a)	22.03 ± 4.26 (a)
2 year	2–1	15.9	8.84	0.85	21.28
	2–2	17.45	11.07	1.23	37.91
	2–3	14.6	8.63	0.88	21.30
	2–4	15.8	8.37	0.88	20.78
	Mean ± S.D.	15.94 ± 1.17 (a)	9.22 ± 1.24 (a)	0.96 ± 0.18 (a)	25.32 ± 8.40 (a)
3 year	3–1	15.3	10.05	0.93	26.70
	3–2	18.1	12.03	1.20	40.93
	3–3	14.1	8.79	0.82	20.43
	3–4	18.2	12.42	1.10	39.05
	Mean ± S.D.	16.43 ± 2.05 (a)	10.82 ± 1.71 (a)	1.01 ± 0.17 (a)	31.78 ± 9.86 (a)
4 year	4–1	14.35	10.805	1.1555	35.03
	4–2	12.7	8.145	0.9005	20.49
	4–3	16.4	11.32	1.2215	38.75
	4–4	15.7	9.8	0.9795	27.14
	Mean ± S.D.	14.79 ± 1.63 (ab)	10.02 ± 1.40 (a)	1.06 ± 0.15 (a)	30.35 ± 8.16 (a)
5 year	5–1	13.45	7.79	0.7	15.59
	5–2	12.2	8.455	1.021	23.85
	5–3	11.7	8.475	0.9035	21.49
	5–4	12.5	8.19	0.766	17.87
	Mean ± S.D.	12.46 ± 0.74 (b)	8.23 ± 0.32 (a)	0.85 ± 0.14 (a)	19.70 ± 3.68 (a)

*Note:* For each group, individual sample values are listed followed by the mean ± standard deviation (S.D.). Different lowercase letters within a column indicate significant differences among age classes (one‐way ANOVA followed by Tukey's multiple comparison test, *p* < 0.05); means that share the same letter are not significantly different.

### 2.2 Sap Flux Density Measurement

The sap flux density in all bamboo samples was measured using the Granier method, which is widely used to estimate transpiration in bamboo forests (Granier 1987; James et al. 2002; Kume et al. 2010; Ichihashi et al. 2015; Yang et al. 2015). To reduce short‐term meteorological noise when comparing culm ages, all sap flux measurements were performed concurrently at the same site during a single late‐summer window (5–12 September 2021). Each sap flow sensor consisted of two thermocouple probes, each 2 mm in diameter and 10 mm in length (James et al. 2002; Kume et al. 2010), which were inserted approximately 7 mm into the culm wall, and calibration was performed accordingly. The two probes were inserted into the bamboo culm at heights of approximately 1.15 and 1.3 m above ground, spaced 15 cm vertically apart in the xylem tissue. The upper probe contained a heater supplying a constant power of 0.15 W (James et al. 2002; Kume et al. 2010), and the temperature difference induced by the sap flow was recorded.

Temperature differences were logged every 30 s using a CR1000 data logger and an AM16/32 multiplexer (Campbell Scientific Inc.) and averaged at 30 min intervals. The sap flow velocity (u) was calculated according to Granier (1987) and James et al. (2002), using Equation (2):

(2) $$u=1.19\times 10^{-4}{\left(\frac{∆T_{\max}-∆T_{u}}{∆T_{u}}\right)}^{1.23}$$

where $∆T_{\max}$ is the maximum daily temperature difference, and $∆T_{u}$ is the temperature difference at a given measurement time.

Multiplying the average sap flow velocity (u) by the culm cross‐sectional area ($S_{A}$) yielded the total sap flow rate (*F* (Equation (3))).

(3) $$F=u\times S_{A}$$

### 2.3 Dye Injection Experiment

After completing the sap flux measurements, a dye‐injection experiment was conducted on the 20 bamboo culms. Following previous studies (Sano et al. 2005; Umebayashi et al. 2007, 2008, 2010; Kume et al. 2010), a funnel was placed 1.4 m above ground on standing bamboo. A 0.2% acid fuchsin aqueous solution (Umebayashi et al. 2008; Xiang et al. 2023) was added to the funnel. While the funnel contained the dye, a hand saw was used 1.5 m above ground to make a transverse cut in the bamboo culm. After the cut was established, the dye injection proceeded via the cut for 30 min (based on a preliminary experiment, *n* = 3; the mean ascent time to crown was about 30 min).

After the dye injection, the sample bamboo was removed from the base, and the culm was immediately frozen in liquid nitrogen to fix the dye. Culm discs, each 5 cm in thickness, were collected at 30 cm (1.8 m height), 40 cm (1.9 m height), and 50 cm (2.0 m height) above the dye injection point and stored in liquid nitrogen to prevent further dye diffusion.

All culm discs collected at 1.8 m were freeze‐dried to fix the dye (FDU‐2200; EYELA, Japan). The discs were then subdivided into 1.5 cm‐wide blocks, and both cross‐sections and longitudinal sections were prepared using a sliding microtome (IVS‐410, IKEDA, Japan). Finally, the sections were examined and photographed under a stereomicroscope (SZM223B, AS ONE, Japan) to observe dye distribution.

### 2.4 Leaf Area Measurement

After the dye injection experiment, all leaves were collected from each sample bamboo. In early October 2021, 25 leaves were randomly selected from each sample and scanned (600 dpi, GT‐X830, EPSON), and the leaf area was calculated using image analysis software (Scion Image, Scion Corp., Huang et al. 2019). The scanned leaves were then dried in an oven (70°C, 48 h; EO‐600 V, ETTAS) and weighed to determine dry mass. The total leaf area was estimated using the regression relationship shown in Equation (4).

(4) $$S_{L}=\frac{S_{\mathrm{LA}}}{{\mathrm{DW}}_{A}}\times \mathrm{DW},$$

where $S_{L}$ is the total leaf area for each sample, $S_{\mathrm{LA}}$ is the scanned leaf area, ${\mathrm{DW}}_{A}$ is the dry mass of the scanned leaves, and DW is the total dry mass of all leaves collected from each sample.

### 2.5 Water Distribution Observation and EDS Analysis

To minimize freezing artifacts, culm discs excised at 2.0 m height were immediately plunge‐frozen in liquid nitrogen for 2 min, transferred to an insulated cool box pre‐chilled with reusable ice packs (Keep Thermo Ice KTI‐51, −51°C, Sunyou Printing Co. Ltd.), and transported to the laboratory, where they were stored in a deep freezer at −60°C until analysis (Xiang et al. 2024). Each disc was subdivided into 1.5 cm‐wide blocks, which were trimmed at −20°C using a cryostat microtome (OLAR‐D, Sakura Finetek). The blocks were then transferred under liquid nitrogen to a cryo‐scanning electron microscope (JSM‐IT200, JEOL) to observe water distribution using an accelerating voltage of 5 kV. Photographs were taken to document water distribution (Utsumi et al. 1998, 1999, 2003; Xiang et al. 2023).

An energy‐dispersive X‐ray spectroscopy (EDS) detector attached to the same cryo‐scanning electron microscopy (SEM) system (Tihlaříková et al. 2019) was used to measure the mass percentages of K and Si in the vessel sap of each bamboo sample.

### 2.6 Relative Loss of Potential Conductivity (RLPC)

Cryo‐SEM images of each bamboo sample were examined to measure the major axis (a) and minor axis (b) of each vessel lumen in ImageJ software (1.52 k, National Institutes of Health, Bethesda, Maryland, USA). The mean vessel diameter (D) was then calculated (Lewis and Boose 1995; Xiang et al. 2023) according to Equation (5):

(5) $$D=\frac{2a^{3}b^{3}}{a^{2}+b^{2}}.$$

Subsequently, the theoretical hydraulic conductivity ($K_{n}$) of the bamboo vessels was derived from the Hagen–Poiseuille law (Tyree and Zimmermann 2002), as shown in Equation (6) (Hirose et al. 2005; Xiang et al. 2023):

(6) $$K_{n}=\frac{D^{4}\pi \rho \;}{128\mathrm{\eta W}\;},$$

where D is the mean vessel diameter (cm), ρ is the density of water at 25°C (g·cm^−3^), and ηW is the viscosity of water (g cm^−1^ s^−1^).

On a per‐unit cross‐sectional area basis, the total theoretical hydraulic conductivity of the embolized vessels ($K_{n\_\text{embo}}$) was compared with the total theoretical hydraulic conductivity of all vessels ($K_{n\_\mathrm{all}}$). This ratio was used to obtain the relative loss of potential conductivity (RLPC), as shown in Equation (7).

(7) $$\text{RLPC}=\frac{K_{n\_\text{embo}}}{K_{n\_\mathrm{all}}}\times 100\%.$$

RLPC is defined as the ratio of the hydraulic conductivity theoretically contributed by the embolized vessels to the total theoretical conductivity. This metric represents the proportion of potential water transport capacity that is assumed to be lost owing to embolism, under the assumption that all vessels were initially functional.

### 2.7 Starch Distribution Observation

All culm discs collected 1.9 m above ground were preserved in FPA fixative (Johansen 1940). In mid‐October 2021, each disc was cut into 1 cm‐wide blocks, which were then embedded in LR White resin (Andriotis et al. 2010). Embedded samples were sliced into 12 μm‐thick cross sections using a rotary microtome (OSK 97LF506; OGAWASEIKI). The sections were double‐stained with 0.01% toluidine blue and 1.8% iodine solutions (Andriotis et al. 2010) and then mounted using BioLite. All sections were observed and photographed under a light microscope (Eclipse E600, Nikon).

### 2.8 Statistical Analysis

All statistical analyses were performed using SPSS Statistics version 27 (IBM Corp.). The culm was the independent sampling unit (*n* = 4 per age group); where repeated subsamples existed within a culm (e.g., multiple measurements or multiple vessels), measurements were first averaged at the culm level, and the culm mean was used for analysis. One‐way analysis of variance (ANOVA) followed by Tukey's honestly significant difference (HSD) test was used to examine differences among the age groups (*p* < 0.05). Data visualization was performed using Origin 2021 software (OriginLab Corporation).

## 3 Results

### 3.1 Sap Flux Density and Average Daily Sap Flow Rate

**FIGURE 2:**
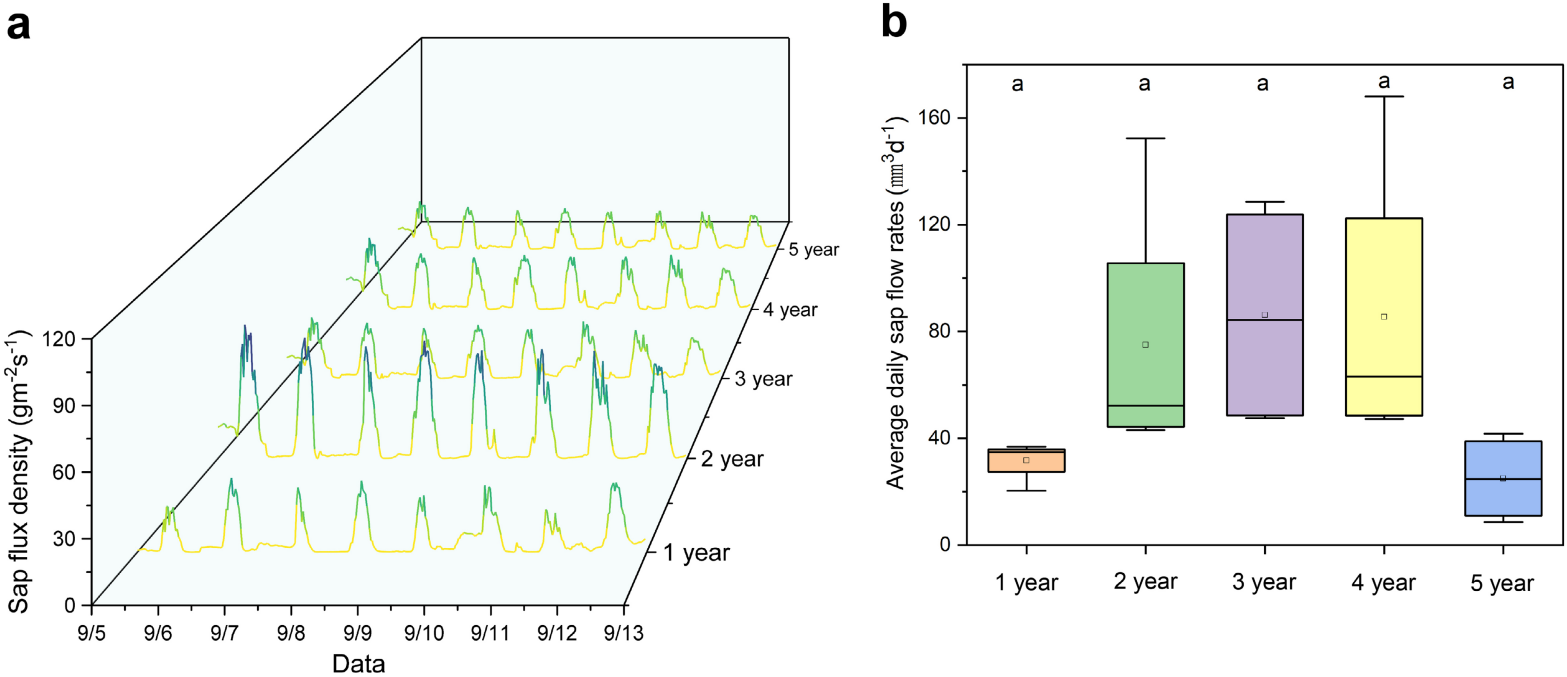
Sap flux density and average daily sap flow rate of Moso bamboo culms across five age groups. (a) Diurnal variation in sap flux density recorded from September 5 to 12, 2021. Colors represent relative sap flux density, with gradients from yellow (low) to green and blue (high). Curves represent representative individual culms per age group. (b) Average daily sap flow rates for culms in different age groups. Data were calculated from four replicates per group (*n* = 4). No statistically significant differences were detected among groups (Tukey's honestly significant difference test, *p* > 0.05).

Based on the calculated data, the 2‐year‐old culm group exhibited a relatively higher sap flux density than the other age groups. No significant differences in sap flux density were observed among the 1‐, 3‐, and 4‐year‐old groups, whereas the 5‐year‐old group showed a slightly lower sap flux density than the other groups (Figure 2a).

The average daily sap flow rate was estimated by combining the cross‐sectional area of each culm with the average sap flux density. Although the 2‐, 3‐, and 4‐year‐old groups exhibited higher daily sap flow rates than the 1‐ and 5‐year‐old groups, statistical analysis (ANOVA) indicated that the differences in daily sap flow rates among the age groups were not statistically significant (*p* > 0.05, Figure 2b).

### 3.2 Average Leaf Area

**FIGURE 3:**
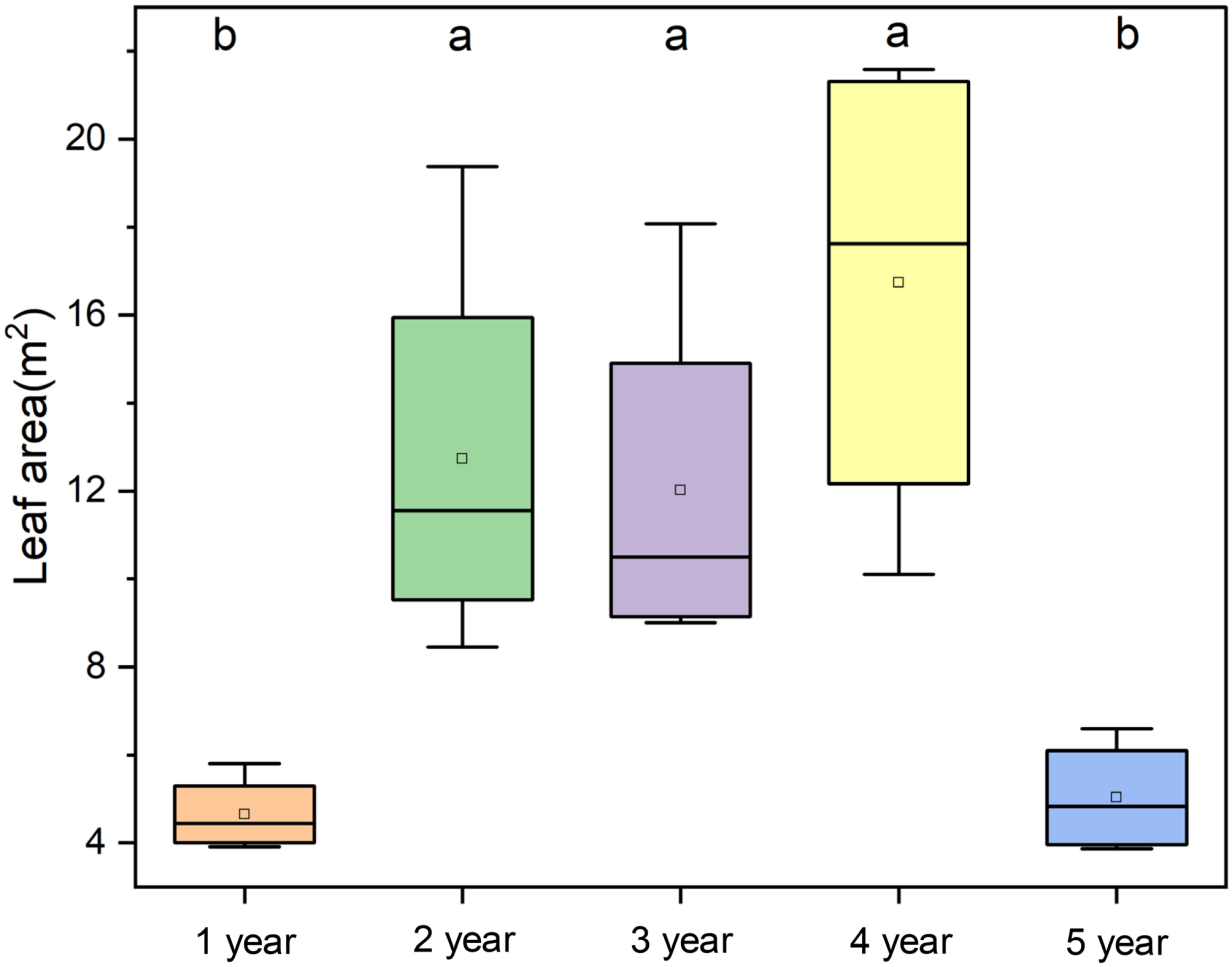
Average leaf area of Moso bamboo culms across five age groups. Data were calculated from four replicates per group (*n* = 4). Different letters above boxes indicate statistically significant differences among groups based on Tukey's honestly significant difference test (*p* < 0.05); shared letters denote non‐significant differences (*p* > 0.05).

The 2‐, 3‐, and 4‐year‐old groups exhibited relatively large average leaf areas, whereas 1‐ and 5‐year‐old groups exhibited small leaf areas. The results of one‐way ANOVA followed by Tukey's HSD test indicated that the 2‐, 3‐, and 4‐year‐old groups had significantly larger leaf areas than the 1‐ and 5‐year‐old groups (*p* < 0.05; Figure 3).

### 3.3 Dye Distribution Observation

**FIGURE 4:**
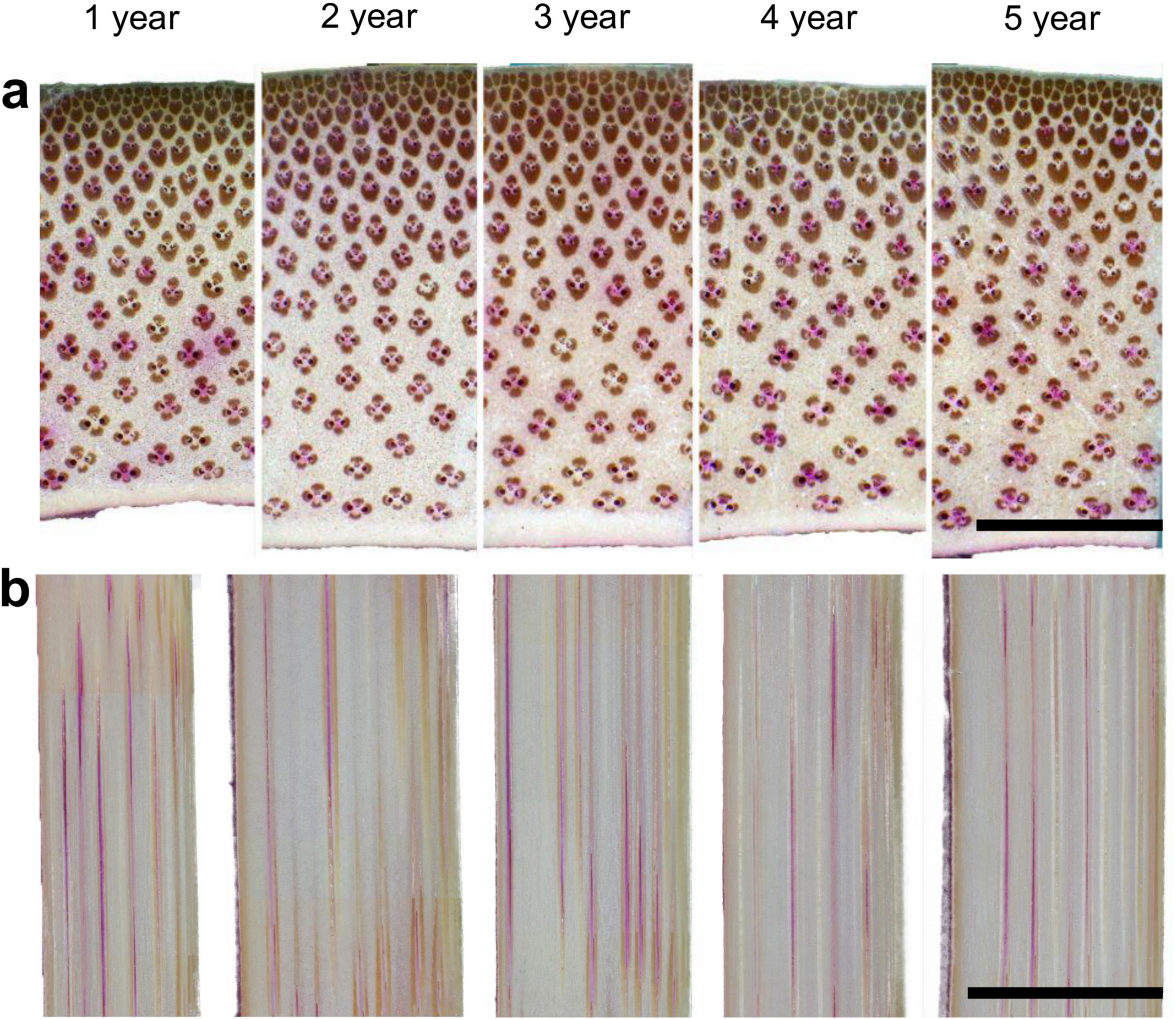
Dye distribution in culms of Moso bamboo across five age groups. (a) Cross‐sectional images of dye‐stained culm discs observed under a stereomicroscope. The upper side of each image corresponds to the bark side, and the lower side to the inner side. Scale bar = 4 mm. (b) Radial longitudinal sections of dye‐stained culms observed under a stereomicroscope. The left side of each image corresponds to the bark side, and the right side to the inner side. Scale bar = 10 mm.

Cross‐sectional observations showed that the dye was continuously distributed within the vessel elements from the outer to inner regions of the culm in all samples. No obvious differences in the pattern of stained vessels were observed among the age groups (Figure 4a). In addition, longitudinal observations confirmed long‐distance dye transport, indicating that axial conductivity was maintained across all age groups (Figure 4b).

### 3.4 Water Distribution and RLPC

**FIGURE 5:**
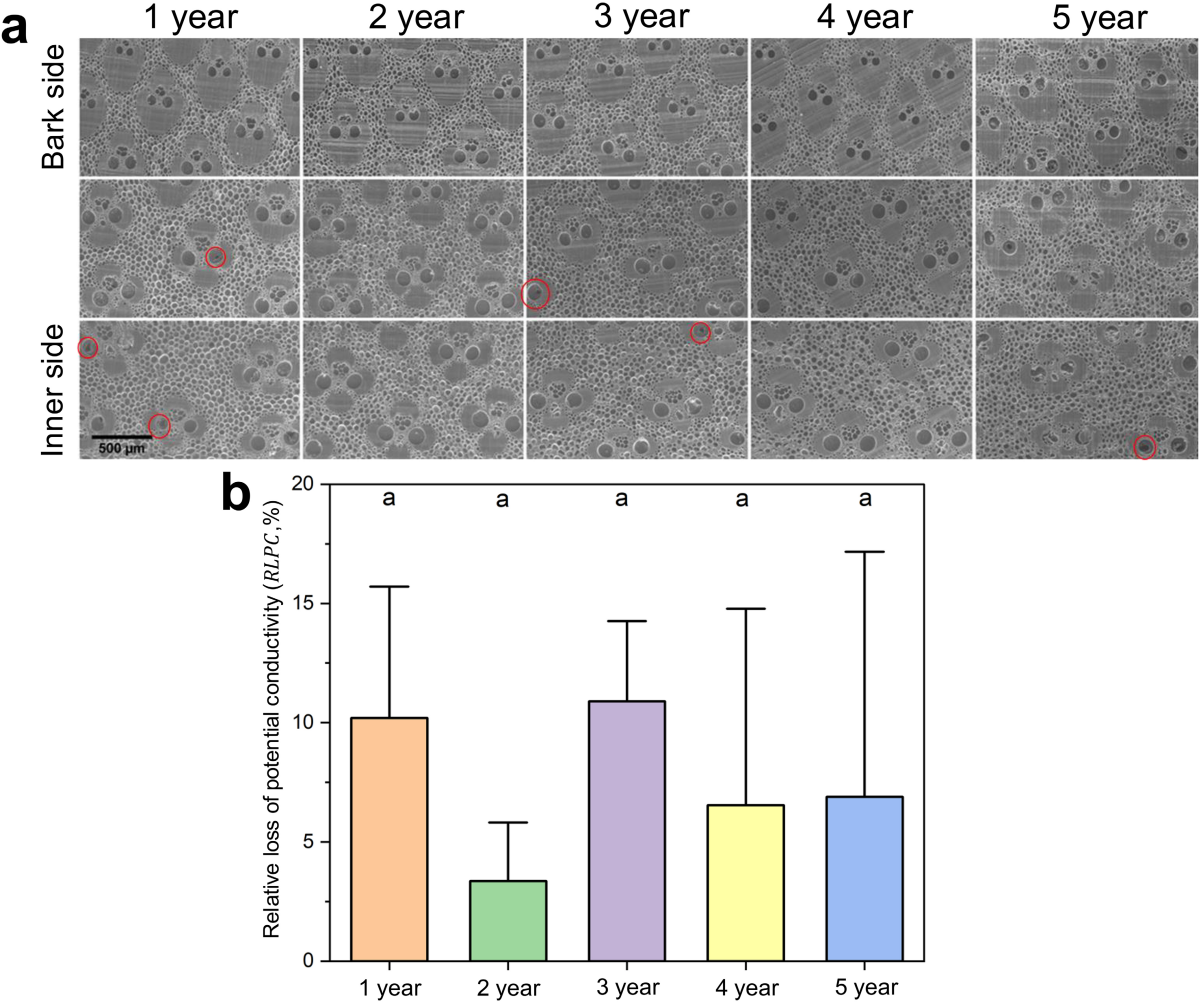
Cryo‐SEM observations and relative loss of potential conductivity (RLPC) in culms of Moso bamboo across five age groups. (a) Representative cryo‐scanning electron microscopy (cryo‐SEM) images of xylem tissue from each age group. For each group, three radial positions from the bark side to the inner side of a single culm are shown. Red circles indicate vessel elements where embolism was observed. (b) RLPC across different age groups. Bars represent the mean ± standard deviation (S.D.), calculated from four replicates per group (*n* = 4). No statistically significant differences were found among groups (Tukey's honestly significant difference test, *p* > 0.05).

Cryo‐SEM cross‐sections revealed that most vessel lumens in all age groups were water‐filled across the radial sections, with embolisms observed only in a few vessels. No increase in the embolism frequency was associated with an increase in culm age (Figure 5a).

The RLPC was calculated for each group to further assess hydraulic obstruction. RLPC values remained generally low across all samples, with a maximum of approximately 10%, and no significant differences were detected among age groups based on one‐way ANOVA (*p* > 0.05; Figure 5b), suggesting a minimal age‐related decline in conductive capacity.

### 3.5 Mass Percentages of Si and K in Vessel Sap

**FIGURE 6:**
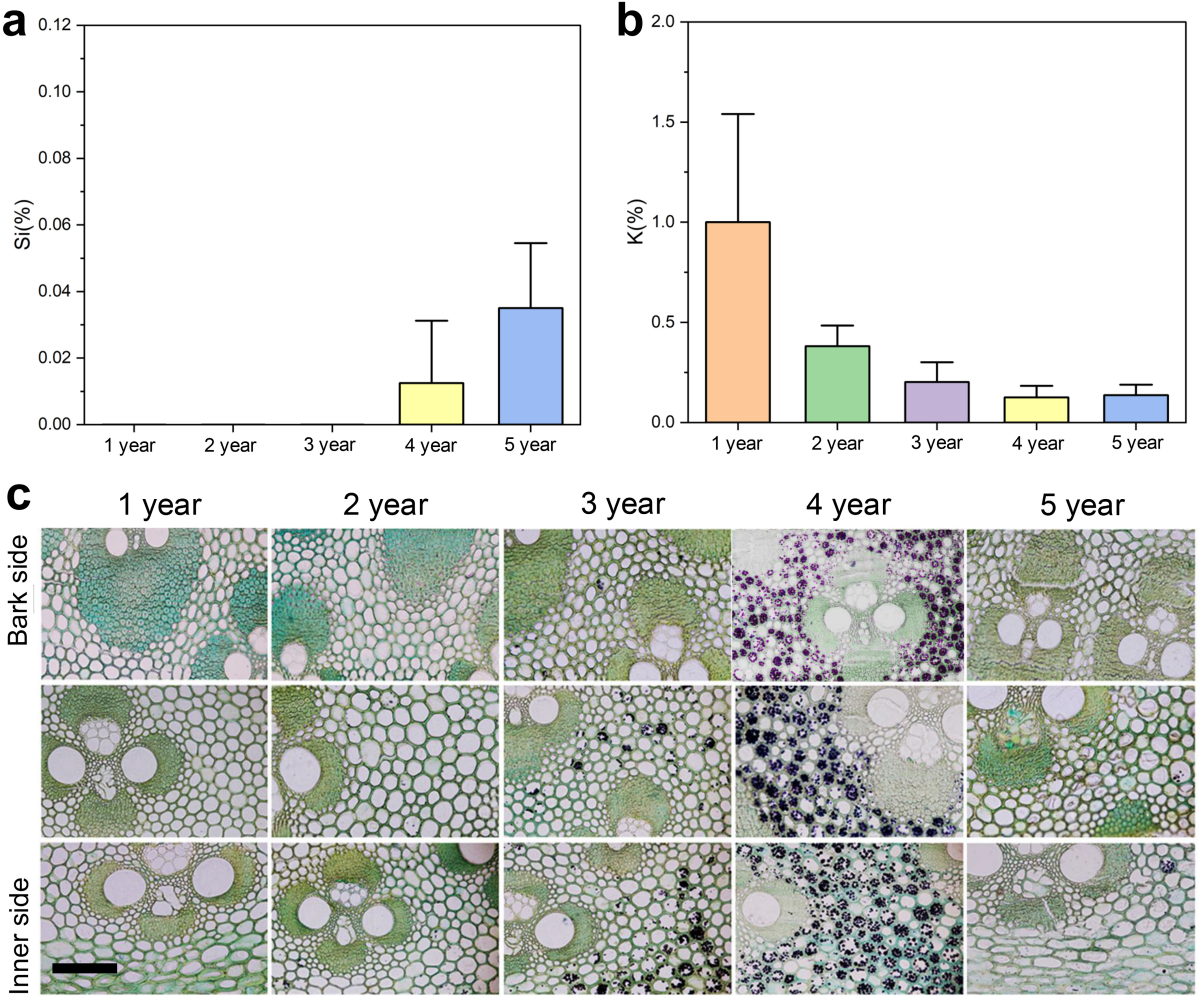
Ion concentrations in vessel sap and starch distribution in parenchymal tissue of Moso bamboo culms across five age groups. (a) Relative concentration of silicon (Si) in vessel sap. (b) Relative concentration of K in vessel sap. Both (a) and (b) were measured using energy‐dispersive X‐ray spectroscopy. Values are expressed as mass percent and normalized to the sum of detected elements (Σ = 100%). Bars represent the mean ± standard deviation (S.D.), calculated from four replicates per group (*n* = 4). (c) Light micrographs of starch distribution in parenchymal cells of representative culms from each age group. For each group, three radial positions from the bark side to the inner side are shown. Dark‐stained regions in parenchymal cells indicate the presence of stained starch granules. Scale bar = 100 μm.

Simultaneously with the cryo‐SEM observations of water distribution, the mass percentages of Si and K in the vessel sap were measured using an EDS detector attached to the cryo‐SEM. Si was not detected in the vessel sap of the 1‐, 2‐, and 3‐year‐old bamboo samples (below the detection limit), whereas a low mass percentage of Si was detected in the 4‐year‐old group, which increased further in the 5‐year‐old group (Figure 6a).

By contrast, the mass percentage of K was relatively high in the 1‐year‐old group and decreased annually thereafter. In the 4‐ and 5‐year‐old groups, K content remained relatively low and stable (Figure 6b).

### 3.6 Starch Distribution Observation

No obvious starch accumulation was observed in the parenchymal cells of the 1‐, 2‐, and 5‐year‐old groups. In the 3‐year‐old group, starch accumulation began to appear in the parenchymal cells from the outer to inner regions of the culm. In the 4‐year‐old group, nearly all parenchymal cells throughout the cross‐section exhibited strong starch accumulation, as indicated by the characteristic blue‐black staining (Figure 6c).

## 4 Discussion

### 4.1 Hydraulic Performance and Its Coordination With Leaf Area Across Culm Age

In this study, two‐year‐old Moso bamboo culms exhibited the highest sap flux density (Figure 2a). This trend is likely attributable to the fact that after 1 year of rapid growth, culms typically reach a stage of structural maturity in the second year, characterized by increased tissue density and thickening of parenchymal cell walls (Uchida et al. 2022). These anatomical developments possibly supported the formation of fully developed vessel elements and enhanced hydraulic efficiency. Consequently, the water transport efficiency per unit xylem area peaked during this stage. A slight decline in hydraulic efficiency possibly occurred from the third year onward (Figure 2a). In other woody species, age‐related reductions in water transport efficiency have been associated with the accumulation of deposits in the vessel lumen and thickening of vessel walls, both of which can reduce the effective lumen diameter (Alvin and Murphy 1988; De Boer and Volkov 2003; Wahab et al. 2010). Although these mechanisms were not directly examined in the present study, they may provide a physiological context for interpreting the observed trends.

In this study, two‐year‐old Moso bamboo culms showed the highest sap flux density among the age classes (Figure 2a). However, their daily sap flow was broadly comparable to that of the other age classes, and overall differences in whole‐culm water use were not statistically significant (Figure 2b). To understand why higher sap flux density did not translate into greater whole‐culm water use, it is necessary to consider water transport capacity at the scale of the entire culm, which depends on both sap flux density and the cross‐sectional area of conductive tissue. Mean conductive area tended to be larger in three‐ and four‐year‐old culms and smaller in one‐ and five‐year‐old culms, but these differences were not statistically significant among age classes (*p* > 0.05; Table 1), consistent with the absence of pronounced secondary thickening growth in Moso bamboo. Because total sap flow can be regarded as the product of sap flux density and conductive area (Cermak and Nadezhdina 1998), such differences in culm dimensions can partly buffer age‐related variation in sap flux density per unit area, resulting in broadly similar daily sap flow across age classes.

In addition, the trend in the daily sap flow rate closely mirrored that of the total leaf area across age groups (Figures 2b and 3). Culms aged 2–4 years had relatively larger total leaf areas and exhibited higher sap flow rates, whereas those aged 1 and 5 years had smaller leaf areas and correspondingly lower sap flow rates. This pattern suggests a close physiological coordination between transpiration demand at the leaf level and water transport capacity at the stem level. Similar coordination between leaf area and conductive tissue has been reported in plants, where larger leaf areas typically correspond to higher water flux modulated by anatomical and hydraulic traits (Meinzer et al. 2001; O'brien et al. 2004).

Recent studies on woody bamboo have highlighted the potentially important role of nocturnal root pressure in maintaining xylem hydraulic functions. Several studies have demonstrated that positive root pressure can occur during the night and effectively refill embolized vessels, thereby contributing to the nocturnal recovery of hydraulic conductivity (Yang et al. 2012). Similarly, other studies have shown that this root pressure may support night‐time sap flow, particularly under drought conditions, by sustaining water transport from late night to early morning hours (Wang et al. 2011; Yang et al. 2015). Although root pressure was not directly measured in the present study, we observed a slight increase in sap flux density during the night‐to‐dawn period under clear and dry conditions (e.g., from the night of September 9 to the early morning of September 10; Figure 2a). This pattern may suggest the presence of root pressure‐driven water movement, which could help restore vessel functionality and support the overall water transport capacity, especially in older culms.

These findings suggest that a single indicator may not adequately reflect the age‐dependent hydraulic characteristics of Moso bamboo. This study emphasizes the importance of simultaneously considering water transport efficiency per unit area (e.g., sap flux density), whole‐plant functional output (e.g., total sap flow), the regulatory role of leaf area, and potential contributions from root pressure to comprehensively understand the water‐use strategies of bamboo across different developmental stages.

### 4.2 Age‐Related Stability of Water Transport Pathways in Moso Bamboo Culms

The dye distribution results revealed that vessel elements exhibited high axial and radial continuity in the Moso bamboo culms across all age groups (Figure 4). Cross‐sectional observations showed no significant differences in the pattern of stained vessels among culms of different ages (Figure 4a), whereas longitudinal sections further confirmed that the dye could move over long distances through the vessel system (Figure 4b). These findings indicate that the structural and functional integrity of water transport pathways is well preserved in 1‐ to 5‐year‐old culms, with no obvious decline in axial or radial conductivity. These results are consistent with those of a previous study on water transport pathways and dye distribution patterns in Moso bamboo (Kume et al. 2010).

Cryo‐SEM observations further supported the conclusion that the hydraulic structure and function of bamboo culms remain stable across different age stages. In all age groups, most vessel lumens observed in cross‐sections were completely filled with water, and only a few embolized vessels were detected. Moreover, no increasing trend in embolism frequency was observed with increasing culm age (Figure 5a). Quantitative analysis of the relative loss of potential conductivity (RLPC) revealed generally low RLPC values across all groups, with a maximum of approximately 10%, and no significant differences were found among the age classes (Figure 5b). These results indicate that embolism remains infrequent even in 5‐year‐old culms and that the overall distribution of water within the vessel system is stable. The occasional presence of embolized vessels does not appear to compromise the overall hydraulic functionality of the culms.

In summary, evidence from both dye transport experiments and cryo‐SEM imaging strongly supports the conclusion that Moso bamboo maintains high structural durability and functional stability in its vascular system at different ages. Although some age‐related physiological decline may gradually emerge with prolonged growth, the water transport system in culms up to 5 years of age does not show signs of marked deterioration. The sustained structural integrity of the vessel is likely a key anatomical basis for maintaining the high vitality and efficient water use of Moso bamboo across multiple developmental stages.

### 4.3 Physiological Implications of Si and K Accumulation in Vessel Sap and Starch Deposition in Parenchymal Tissue

Elemental analysis of vessel sap using a cryoscanning electron microscope equipped with an EDS system revealed a clear age‐dependent variation in the concentrations of K and Si (Figure 6a,b), indicating their distinct physiological roles in the water transport system and developmental status of bamboo culms. Potassium, a key ion involved in osmotic regulation and the maintenance of cell turgor (Talbott and Zeiger 1996; Johnson et al. 2022; Mostofa et al. 2022), exhibited a relatively high mass percentage in 1‐year‐old culms, followed by a gradual decline with increasing culm age (Figure 6b). This trend likely reflects the vigorous metabolic activity and high transpiration demand during the early growth stages of Moso bamboo, when rapid cell division, xylem differentiation, and internode elongation occur. Potassium facilitates water movement and stomatal regulation during this phase (Roelfsema and Hedrich 2005; Cochrane and Cochrane 2009). As growth stabilizes and xylem tissues mature in later developmental stages, the need for osmotic adjustment decreases. Moreover, K in older culms may be redistributed through the rhizome system and translocated to younger, rapidly growing culms with higher nutrient demand, resulting in a noticeable reduction in K content (Umemura and Takenaka 2014).

By contrast, Si was undetectable (below the detection limit) in the vessel sap of 1‐ to 3‐year‐old culms but began to appear in the 4‐year‐old group and further increased in the 5‐year‐old group (Figure 6a). This pattern suggests that during the early to middle stages of development, Si may be preferentially utilized in actively expanding leaves rather than accumulating in the culm, which is consistent with previous reports indicating a high Si content in bamboo foliage (Collin et al. 2012). However, Si may accumulate in older culms and play a structural role. Moreover, Si enhances mechanical rigidity and may strengthen vessel walls, thereby reducing the risk of collapse or embolism under hydraulic or mechanical stress (Wang et al. 2021). The contrasting patterns of K and Si concentrations may reflect a shift in functional strategy from dynamic water transport support in younger culms to structural maintenance in older culms.

Starch staining of parenchymal tissues further supports the age‐dependent physiological transition (Liese and Weiner 1996; Uchida et al. 2022). No significant starch accumulation was observed in 1‐, 2‐, or 5‐year‐old culms. By contrast, starch began accumulating in the 3‐year‐old group, appearing first in the inner parenchyma and then expanding outward. In the 4‐year‐old group, nearly all parenchymal cells throughout the cross‐section exhibited strong starch accumulation (Figure 6c). These results suggest that starting from the third year, a portion of photosynthates is increasingly allocated to storage, indicating a decline in structural growth demand and a physiological shift toward energy reserve accumulation to support long‐term maintenance or future regrowth of new shoots from the same rhizome. The subsequent disappearance of starch in the 5‐year‐old group may indicate that stored carbohydrates were mobilized and potentially translocated through the rhizome to support the growth of younger, more active culms. Alternatively, this pattern may reflect reduced photosynthetic capacity or the onset of a physiological decline in aging culms.

The dynamic changes in K and Si concentrations and the sequential pattern of starch accumulation across culm ages revealed the coordinated regulation of water transport and metabolic processes in Moso bamboo. The decrease in K concentration and increase in Si concentration may reflect a functional transition from active hydraulic performance to structural reinforcement. At the same time, starch accumulation is an important indicator of the shift toward physiological maintenance and energy storage. These anatomical and biochemical features indicate culm maturity and functional status, offering novel insights into water transport strategies and age‐related adaptation mechanisms in perennial monocotyledonous species.

### 4.4 Functional Transitions and Long‐Term Water‐Use Strategy in Moso Bamboo: Implications for Invasion Ecology and Forest Management

Although no significant differences in overall water use were observed among culms of different ages, clear age‐dependent functional transitions in elemental (K and Si) and starch allocation strategies were identified. Early‐ and middle‐aged culms exhibited higher hydraulic efficiency, larger transpiring leaf areas, and higher K content in the vessel sap, supporting active transpiration and vigorous growth. By contrast, although hydraulic efficiency did not substantially decline in the later stages, Si accumulation and starch storage in the parenchymal tissues significantly increased, suggesting a shift in physiological function toward structural stabilization and energy storage. These results indicate that while the water transport efficiency remains relatively stable throughout the different developmental stages, Moso bamboo achieves a functional balance between short‐term resource allocation and long‐term physiological sustainability through nutrient strategy adjustments.

This coordinated functional shift enhanced the physiological resilience of Moso bamboo and may constitute an important ecological basis for its successful expansion beyond its native distribution range. Notably, even in the fifth year, culms maintained low embolism levels and stable structural integrity of vessel elements, suggesting that hydraulic function can be stably maintained over an extended lifespan. This stability allows bamboo to retain strong transpiration and water uptake capacity even under stressors such as seasonal drought or interspecific competition.

These robust hydraulic properties may confer competitive advantages over native tree species, particularly in mixed or plantation forests with limited water resources. The combination of high early‐stage water‐use efficiency and sustained transport capacity in older culms enables bamboo to effectively occupy forest edges and understories. Moreover, the functional transition from K‐based osmotic regulation to Si‐based structural reinforcement suggests that mature culms gradually shift from active resource absorption to a supportive role, reallocating nutrient resources to expand new culms and rhizomes.

### 4.5 Limitations and Outlook

Our study intentionally concentrated all measurements within a single late‐summer window (5–12 September 2021) at one site to minimize meteorological noise for age comparisons; subsequent laboratory analyses (e.g., dye‐tracing imaging, cryo‐SEM/EDS, and starch staining) were performed on these field‐collected materials. As a result, inter‐annual and seasonal variability—as well as potential cohort effects—were not captured. The age‐related differences reported here should be interpreted as late‐summer patterns for this site. In future work, we plan to conduct multi‐season and multi‐site monitoring over a one‐year period to disentangle seasonal dynamics (e.g., in K and Si concentrations and starch deposition) from pure age effects, thereby strengthening the generality of the findings.

## 5 Conclusion

This study revealed pronounced age‐dependent plasticity in the hydraulic and metabolic traits of Moso bamboo culms, which may serve as a key physiological basis for the ecological adaptability and invasive potential of this species. The K concentration in vessel sap was highest in 1‐year‐old culms and progressively declined with age, whereas culms aged 2–4 years exhibited higher sap flux density and larger leaf areas. From the third year onward, bamboo maintained a stable water transport capacity while gradually accumulating Si and storing starch in parenchymal tissues. These patterns suggest a functional transition from a “growth phase” to a “maintenance phase,” enabling bamboo to sustain physiological activity and population renewal under complex environmental conditions. From a management perspective, understanding age‐related water‐use strategies in Moso bamboo offers practical guidance for controlling its spread. The selective removal of middle‐aged culms with large transpiring leaf areas and high water demand while retaining structurally stable and metabolically conservative older culms may help reduce total water consumption and mitigate bamboo encroachment into adjacent stands. In addition, physiological markers such as vessel integrity, Si accumulation, and starch distribution could serve as reliable indicators of culm maturity and functional status, providing a scientific basis for age‐structure‐informed monitoring and management strategies.

## Author Contributions

Y.X. and Y.U. designed the study and conducted all the experiments. S.K. supported the sample selection and collection. T.K. supported the sample selection and sap flux density measurements. S.N. supported the cryo‐SEM analysis. M.Y. supported the leaf area measurements. All the authors contributed to the writing of the manuscript and approved the submitted manuscript.

## Conflicts of Interest

The authors declare no conflicts of interest.

## Data Availability

All data and materials supporting the findings of this study are available from the corresponding author upon reasonable request.
